# Self-reflection and screening mental health on Canadian campuses: validation of the mental health continuum model

**DOI:** 10.1186/s40359-020-00446-w

**Published:** 2020-07-29

**Authors:** Shu-Ping Chen, Wen-Pin Chang, Heather Stuart

**Affiliations:** 1 Department of Occupational Therapy, Faculty of Rehabilitation Medicine, University of Alberta, 2-30 Corbett Hall, Edmonton, Alberta T6G 2G4 Canada; 2 Indiana University (IUPUI), Indianapolis, Indiana USA; 3 Queen’s University, Kingston, Ontario Canada

**Keywords:** Mental health continuum, Prevention, de-stigmatization

## Abstract

**Background:**

This study describes the psychometric testing of the Mental Health Continuum (MHC) model the Canadian Department of National Defense developed initially, among undergraduates of three Canadian universities. The MHC is a tool that consists of 6 items to guide students the way to attend to, or monitor, signs and behavior indicators of their mental health status and suggest appropriate actions to improve their mental health.

**Methods:**

Online survey data were collected from 4206 undergraduate students in three universities in two Canadian provinces during the spring of 2015 and winter of 2016. Participants completed an online survey questionnaire that consisted of the MHC questionnaire, the Kessler Psychological Distress Scale (K-10), and demographic information, including age, gender, and year of study.

**Results:**

Factor analysis using the principal components method followed by a two-step internal replication analysis showed that the MHC tool was two-dimensional and that all six domains assessed were crucial. The construct (convergent) validity of the MHC tool was tested against the K-10, and the correlation analysis results were strong overall, as well as within subgroups defined by gender, year of study, and university.

**Conclusions:**

The MHC is a useful tool that helps college students reflect on and enhance their mental health.

## Background

Mental health issues are a growing public health concern in Canada [1–3]. For example, it has been estimated that nearly 355,000 working Canadians would not be unable to work in any given week as a result of mental illnesses or mental and behavioral disorders [4]. In a survey conducted in October 2015, 66% of surveyed employees (*n* = 1023) across Canada who took time off work because of mental health conditions did not report it officially [5]. Further, the estimated expenditure on non-dementia-related mental healthcare was $15.8 billion in 2015 [6], and still, one-third of Canadians aged 15 or older may not have their mental healthcare needs met fully [7]. In particular, more than half of Canadians who experience major depression, one of the most prevalent conditions, may fail to receive satisfactory care [8]. Mental illness has become one of the leading causes of disability in Canada [9].

Managing mental health issues continues to be a critical topic, not only in Canada but worldwide. A growing body of international research has demonstrated the positive effects of mental health promotion, prevention, and early intervention on ameliorating mental health problems and increasing the return on investments [10–18]. For example, Friedli and Parsonage [12] found that, in the United Kingdom, early intervention prevented children from developing conduct disorders that resulted in lifetime savings of £230,000 per child. Arango et al. [10] reviewed ample studies and found that preventive mental health strategies may reduce the incidence of mental health disorders or debilitating outcomes. The authors also highlighted the importance of improving early detection in clinical settings, schools, and the community.

The first step necessary to improve the accuracy of early detection and prevent mental health problems is to screen carefully for early signs or symptoms. Currently, several validated tools are used to screen mental status and monitor symptom severity across treatment for generic symptoms of mental ill-health, as well as specific mental health conditions. Examples of such tools include the Kessler Psychological Distress Scale (K10) [19], Health Questionnaire-28 (GHQ-28) [20], The Beck Depression Inventory (BDI) [21], the Post-Traumatic Stress Diagnostic Scale (PDS) [22], the nine-item Patient Health Questionnaire (PHQ-9) [23], and the Generalized Anxiety Disorder seven-item GAD-7 [24]. Although these screening tools are recognized well, they are not typically available to the general public. With respect to assessment instruments available for use in the public sector, Jensen-Doss and Hawley [25] specified that they must be brief, free or low cost, validated for use, and easy to administer, score, and interpret. Public health researchers have echoed these recommendations and suggested that they must be crucial to patients, easy to administer, and actionable for patients who use them [26]. Therefore, to help the general public assess and monitor their mental health status regularly, it is important to have a simple tool that is easy to understand and administer.

**Fig. 1:**
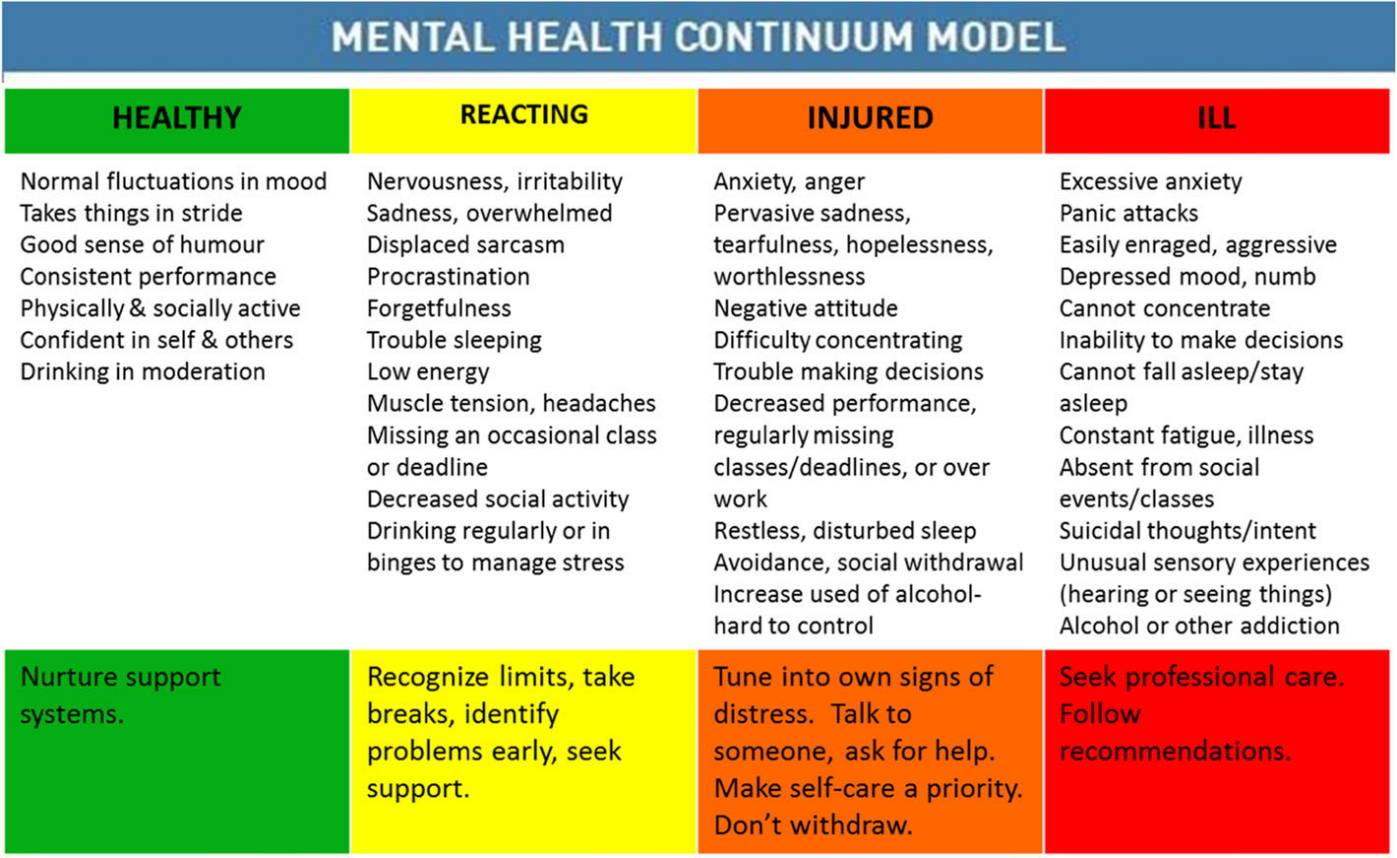
Mental Health Continuum Model (Source: Canadian Armed Forces)

The Department of National Defence Canadian Armed Forces developed the Mental Health Continuum (MHC) model to demonstrate that an individual’s mental health status ranges on a continuum [27]. This model focuses on six major areas—mood, attitude and performance, sleep, physical symptoms, social behavior, and alcohol and gambling—each of which identifies specific mental conditions and challenges along the continuum. This model is intended to serve as a self-reflection and self-monitoring tool “… to teach people to look for signs and behavioural indicators in themselves and others, and to take appropriate actions when they appear. Colours designate levels of severity, bypassing diagnostic labels and the stigma attached with them” (p. S16) [28]. Visually, the MHC Model is composed of four color blocks (green, yellow, orange, red) on a sliding scale from left to right (see Fig. 1). The key features of this model are that it avoids jargon and uses common, destigmatizing language about the risk of mental health conditions by referring to one’s mental health status as “green,” “yellow,” “orange,” or “red” on the color spectrum. The intention is to promote recognition and facilitate and encourage conversation about mental health problems among help-seekers and health professionals (e.g., I feel “yellow” today). Further, the color spectrum contains recommendations to promote mental wellness. A strength of the continuum is that the arrow under the four color blocks conveys the idea that one can move bi-directionally along the continuum. Thus, it is always possible for a person to shift his/her mental health status toward “green,” indicating a return to full mental health and functioning. This continuum model has been adopted widely to promote mental health in various contexts and settings in Canada. However, there is a lack of available studies on its validation in the Canadian postsecondary population.

College and university students are important target populations for mental health screening because they are at high risk of developing mental health problems [29]. The Canadian Association of College and University Student Services found that 18.4 and 14.7% of Canadian postsecondary students, respectively, received a diagnosis or were treated for anxiety or depression during the 2015 academic year [30]. Further, the results of the graduating Canadian university students survey published in 2018 showed that 14% of those surveyed self-identified a mental health disability as the most common disability [31]. More recently, 63.3% of Canadian postsecondary students felt things were hopeless, 88.2% felt overwhelmed, 87.6% felt exhausted, 76.2% felt very sad, and 68.9% felt overwhelming anxiety [32]. These statistics are indicative of significant mental health issues among postsecondary students and the need to address diverse mental health support services on campuses.

Most Canadian postsecondary institutions offer mental health services; however, those offered vary considerably in the range and depth of their support and counseling services [33]. In addition, students understand these campus mental health services poorly, and most pertinent initiatives do not appear to be conducted comprehensively [33, 34]. Hence, additional resources are still required to meet the unfilled needs of student requests [35], and the average wait time to receive campus mental health services in Canada is 19.3 weeks [36]. As a result, this suggests that there are barriers that prevent students from accessing and using services, indicating that many Canadian post-secondary students are left without the support they need.

Research also has shown that college students have developed more negative attitudes toward seeking professional assistance from campus mental health services and tend to avoid them as a result [37, 38]. One of the many reasons for this phenomenon is the stigma associated with mental health problems [39, 40] and the perceived campus culture [41] that precludes many individuals from seeking help [42, 43]. Thus, one approach to address college students’ burden of mental ill-health is to help them expand and build their awareness and self-efficacy. The MHC model may be one useful option to do so. The MHC model emphasizes that people can ***shift*** their mental status toward “green” – not only the absence of mental health problem, but also the presence of positive mental and emotional characteristics. The conception of dynamic mental status in a continuum recognizes that students can be in distress due to life stressor, but those who are emotionally and mentally resilient can cope with difficult situations and able to bounce back from adversity.

However, the MHC model has not been validated comprehensively in the post-secondary student population, and hence, the purpose of this study was to do so to fill this gap. This study was completed originally as part of the Caring Campus Project - a three-year intervention project funded by Movember Canada at three Canadian universities during 2013–2016 [44]. The Caring Campus Project was a health promotion initiative applying an overall participatory framework to help first-year male students increase their awareness with respect to mental health and drinking, reduce stigma attached to substance misuse and to mental health problems, and create a supportive and caring campus environment [41, 44, 45]. The MHC model was one of the tools used as part of the Caring Campus initiative for students to build self-awareness and self-efficacy on their mental health. In this study, we formulated the MHC model in a questionnaire format and investigated its psychometric properties (specifically, the factor structure and evidence for construct (convergent) validity) among Canadian post-secondary students.

## Methods

### Sample and data collection

We conducted a campus-wide survey in three universities - University of Calgary and University of Alberta in Alberta and Queen’s University in Ontario - during the spring of 2015 and winter of 2016. Participants in two of the universities were contacted by email, while those in the third university were recruited through a Research Participation System the Psychology Department hosted. Each university’s Research Ethics Board approved the study procedure, and data were collected from 4206 undergraduate students. The participants provided informed consent and then completed an online survey questionnaire. The questionnaire consisted of the MHC questionnaire, the Kessler Psychological Distress Scale (K-10), and demographic information, including age, year and program of study, and gender. The gender question (how do you identify your gender-Male/Female/Other), rather than a sex question, was specifically asked because gender identity may be more accurately reflect survey respondents’ lives and experience. The demographic data provide useful insights into the composition of participants and their context.

### Measures

#### The mental health continuum

The MHC consists of six items: Mood, Attitude and Performance, Sleep, Physical Symptoms, Social Behavior, and Alcohol and Gambling. Each item has four options and the participants were asked to endorse the one option that reflected best their degree of mental health in the past 30 days. Each item was scored from least severe (1-green) to most severe (4-red), and the scores were summed to provide a total MHC score.

#### The K-10

This scale includes 10 items intended to measure the degree of distress based on questions about anxiety and depressive symptoms that a person has experienced in the most recent 4-week period. Each of the 10 questions is scored 1 (none of the time) to 5 (all of the time) and the scores are summed to provide a total K-10 score. A total score of less than 20 reflects someone who is likely to be well, one of 20–24 reflects a mild mental disorder, one of 25–29 reflects a moderate mental disorder, and a score of 30 and above reflects a severe mental disorder. The K-10 has shown high levels of reliability and validity in various populations [46–48].

### Data analysis

We used SPSS v. 23.0 (IBM Corporation, Armonk, NY) to analyze the MHC data for their factor structure and construct (convergent) validity. To test the MHC’s factor structure, we followed the two-step internal replication analysis Osborne and Fitzpatrick recommended [49]. We split the sample in half randomly and then analyzed each sub-sample separately. Because the items were measured on an ordinal scale, we used the principal components method with varimax rotation for the factor analysis, and the two sub-samples’ factor loadings were compared using the squared differences. Osborne and Fitzpatrick [49] suggested that any item with a squared difference of .04 or greater may need to be omitted because of volatile loadings. To assess convergent validity, we (1) conducted hypothesis testing by evaluating the correlations between the six items’ scores and the total score of the MHC tool, and (2) used the Spearman’s rank-order correlation to determine the correlation between the total score on the MHC tool and that on the K-10.

## Results

### Descriptive data

Table 1 presents the demographic characteristics, K10 score, and MHC score from each university. The participants at each university were predominantly female, among which the largest number attended University B. Because a small proportion of participants indicated their gender identity as “other,” they were excluded from further gender-based analyses. The number of participants was distributed fairly evenly across the 4 years of undergraduate study. The majority of the participants at each university scored “well” on the K-10, while 19.2% overall scored “severe mental disorder.” With respect to the MHC’s item score, the majority of the participants scored less than 2 for any single item, i.e., reacting mental health. Overall, the highest mean score was 1.85 (SD = 1.09) for the Sleep item, while the lowest mean score was 1.30 (SD = 0.55) for the Alcohol and Gambling item. Table 2 further illustrates the distribution of mental statuses for the items of the MHC model at each university. For the first 5 items (mood, attitude, sleep, physical symptoms, and social behaviours), around 40–60% of students were “green-heathy”, 30–50% were “yellow-reacting”, 5–15% were “orange-injured”, and 1–5% were “red-ill”, except for 11–17% of studets had serious disruption in sleep that fall into the category of ill. With regards to alcohol and gambling, 68–79% of students were green, 20–28% were yellow, 2–3% were orange, and less than 1% were red.

**Table 1:** Descriptive results, K10 scores, and scores on the Mental Health Continuum

	University A (*N* = 554)	University B (*N* = 3188)	University C (*N* = 464)	Total (All Combined) (*N* = 4206)
***Gender***				
Male	177 (32.1%)	953 (29.9%)	78 (16.8%)	1208 (28.8%)
Female	371 (67.2%)	2195 (69.0%)	385 (83.2%)	2951 (70.3%)
Other	4 (0.7%)	35 (1.1%)	–	39 (0.9%)
***Year***				
1st	119 (21.5%)	695 (21.8%)	138 (29.7%)	952 (22.7%)
2nd	136 (24.5%)	610 (19.1%)	104 (22.4%)	850 (20.2%)
3rd	153 (27.6%)	701 (22.0%)	95 (20.5%)	949 (22.6%)
4th	100 (18.1%)	652 (20.5%)	76 (16.4%)	828 (19.7%)
5th and up	46 (8.3%)	526 (16.5%)	51 (11.0%)	623 (14.8%)
***K10***				
Well	324 (58.5%)	1350 (42.3%)	245 (52.8%)	1919 (45.6%)
Mild mental disorder	97 (17.5%)	673 (21.1%)	93 (20.0%)	863 (20.5%)
Moderate mental disorder	51 (9.2%)	503 (15.8%)	63 (13.6%)	617 (14.7%)
Severe mental disorder	82 (14.8%)	662 (20.8%)	63 (13.6%)	807 (19.2%)
***Mental Health Continuum:*** mean item score (SD)				
1. Mood	1.57 (0.81)	1.73 (0.81)	1.51 (0.71)	1.69 (0.81)
2. Attitude & performance	1.56 (0.69)	1.77 (0.70)	1.59 (0.64)	1.72 (0.70)
3. Sleep	1.70 (1.00)	1.89 (1.10)	1.74 (1.03)	1.85 (1.09)
4. Physical symptoms	1.67 (0.80)	1.86 (0.84)	1.70 (0.74)	1.81 (0.82)
5. Social behaviours	1.56 (0.72)	1.68 (0.73)	1.47 (0.63)	1.64 (0.73)
6. Alcohol & gambling	1.37 (0.59)	1.30 (0.56)	1.25 (0.49)	1.30 (0.55)

*Note.* K10 score: less than 20 – well; 20–24 – mild mental disorder; 25–29 – moderate mental disorder; 30 and above – severe mental disorder

MHC score: 1 – healthy mental health; 4 – ill mental health

**Table 2:** Distribution of the Mental Health Continuum at each university

	Healthy (Green)	Reacting (Yellow)	Injured (Orange)	Ill (Red)
***Mood***				
University A	273 (58.8%)	152 (32.8%)	29 (6.3%)	10 (2.2%)
University B	1462 (45.9%)	1226 (38.4%)	378 (11.9%)	121 (3.8%)
University C	326 (58.8%)	164 (29.6%)	39 (7.0%)	25 (4.5%)
***Attitude & performance***				
University A	225 (48.5%)	204 (43.9%)	33 (7.1%)	2 (0.4%)
University B	1198 (37.6%)	1557 (48.9%)	397 (12.5%)	36 (1.1%)
University C	303 (54.7%)	194 (35.0%)	54 (9.7%)	3 (0.5%)
***Sleep***				
University A	264 (56.9%)	117 (25.2%)	24 (5.2%)	59 (12.7%)
University B	1598 (50.1%)	857 (26.9%)	214 (6.7)	519 (16.3%)
University C	322 (58.1%)	140 (25.3%)	29 (5.2%)	63 (11.4%)
***Physical symptoms***				
University A	207 (44.6%)	200 (43.1%)	47 (10.1%)	10 (2.2%)
University B	1227 (38.5%)	1342 (42.1%)	470 (14.7%)	149 (4.7%)
University C	280 (50.5%)	194 (35.0%)	61 (11.2%)	18 (3.2%)
***Social behaviours***				
University A	279 (60.1%)	154 (33.1%)	30 (6.5%)	1 (0.2%)
University B	1466 (46.0%)	1317 (41.3%)	350 (11.0%)	55 (1.7%)
University C	312 (56.3%)	177 (32.0%)	59 (10.6%)	6 (1.1%)
***Alcohol & gambling***				
University A	362 (78.0%)	91 (19.6%)	10 (2.2%)	1 (0.2%)
University B	2363 (74.2%)	712 (22.3%)	92 (2.9%)	21 (0.7%)
University C	378 (68.3%)	155 (28.0%)	16 (2.9%)	5 (0.9%)

### Factor structure

Table 3 shows the results of the MHC tool’s factor structure based on the internal replication analysis recommendation. The principal components methods yielded two factors that accounted for 60.78% of the variance in sample 1 (factor 1: 43.76%; factor 2: 17.02%) and 59.15% of the variance in sample 2 (factor 1: 41.96%; factor 2: 17.19%). Both factors had an eigenvalue greater than 1. In both samples, the first factor captured the items of Mood, Attitude and Performance, Sleep, Physical Symptoms, and Social Behavior, and the second captured the item of Alcohol and Gambling. Factor loadings in both samples were similar and strong (all greater than 0.50) for the first and second factors, and the squared differences in the factor loadings for each item between both samples were small.

**Table 3:** Factor Loadings from the Replicability Analysis with Randomly Selected Samples

Item	Sample 1 (*N* = 2124)		Sample 2 (*N* = 2072)		Squared Difference
	Factor 1	Factor 2	Factor 1	Factor 2	
Mood	.79	−.04	.78	.05	.0001
Attitude	.73	−.00	.71	.03	.0004
Sleep	.66	.11	.66	.06	0
Physical	.73	.01	.74	−.01	.0001
Social	.69	−.34	.64	−.34	.0025
Alcohol & Gambling	.19	.95	.14	.95	0
Eigenvalue	2.63	1.02	2.52	1.03	
Variance Explained	43.76%	17.02%	41.96%	17.19%	

### Validity

Significant correlations between the individual items on the MHC tool and the total score were interpreted as evidence of good construct validity in this sample: Mood (Spearman rho = .73, *p* < .001), Attitude and Performance (Spearman rho = .65, *p* < .001), Sleep (Spearman rho = .71, *p* < .001), Physical Symptoms (Spearman rho = .71, p < .001), Social Behavior (Spearman rho = .60, *p* < .001), and Alcohol and Gambling (Spearman rho = .22, *p* < .001). Table 4 presents the results of the analysis of the MHC tool’s construct/convergent validity. The Spearman’s rank order correlations were computed between the total score of the MHC tool’s first factor and the total score of the K-10, as the first factor was related conceptually more to the construct of the K10. The correlations were computed by subgroups based on gender, year of study, site, and the entire sample. The correlation coefficients ranged from .70 to 77 and were robust and statistically significant across gender, year of study, site, and the entire sample. According to Cohen (1988) and Hemphill (2003), correlations of .7 or above would be considered large in terms of magnitude of effect sizes [50, 51]. As shown in Table 4, all of the correlation coefficients were above .7 so considered large.

**Table 4:** Spearman’s Rank Order Correlation of Aggregated Mental Health Continuum Factor 1 Score with the Aggregated K-10 Score

Variable	N	Correlation	*P*-value
Gender			
●Male	1208	.71	<.001
●Female	2951	.74	<.001
Year			
●1	952	.75	<.001
●2	850	.77	<.001
●3	949	.72	<.001
●4	828	.71	<.001
●5+	340	.76	<.001
Site:			
●University A	554	.74	<.001
●University B	3188	.74	<.001
●University C	464	.70	<.001
Overall	4206	.74	<.001

## Discussion

Evidence from this study supported the MHC model’s validity with respect to its factor structure and construct/convergent validity in a large sample of postsecondary students drawn from three universities in Canada. Given the high prevalence of mental health problems among Canadian postsecondary students [29, 32] as well as students in other countries [52, 53], the MHC may provide a beneficial self-assessment and self-monitoring tool to help students maintain optimal mental health.

The results of the factor analysis revealed that the MHC tool loaded strongly on two dimensions. All six item domains were found to be essential, because the factor loadings were above the 0.5 threshold [54]. Interestingly, five item domains loaded together on one factor that represented general distress, and one (the Alcohol and Gambling item) stood as a separate factor. One possible explanation could be that this item is more disorder- and diagnosis-oriented, while the other five item domains are oriented more toward wellbeing. The other explanation could be that these two factors present two different mechanisms that underlie the continuum. The strong convergence between the K-10 total score and the MHC tool total score, both overall and within subgroups defined by gender, year of study, and university site, and the significant (low-to-moderate) correlations between individual item domains and the construct overall (measured by the total score), was interpreted as evidence of validaty in this sample. Overall, the results provide strong preliminary evidence that supports the MHC’s validity and suggests that it may be useful in mental health promotion programs in postsecondary settings. For example, the color spectrum for different MHC domains can help identify areas of strengths and weaknesses in a student’s mental health. Thus, service planning can be tailored to meet each student’s personal needs. We demonstrated that it is also feasible to implement the MHC across different university settings. However, further validation research is still needed to ensure that the tool is useful across a wide spectrum of student populations (e.g., university and college students), and geographic settings.

The MHC model illustrates four different mental health conditons: (1) Healthy - adaptive coping (green), (2) Reacting - mild and reversible distress (yellow), (3) Injured - more severe and persistent functioning impairment (orange), and (4) Ill - clinical illnesses and disorders requiring concentrated medical care (red). The model eliminates the need for stigmatizing labels and non-professionals diagnosing. It emphasizes that mental status is not static and people have possibility to move back and forth along the continuum. Comparing to other evaluation and screening instruments (such as Kessler Psychological Distress Scale-10), the MHC tool not only incorporates signs, indicators, and behavioural cues to identify mental health issues, but also suggest actions to take at each condition of the continuum. The tool provides an excellent structure for health promotion activities. The Canadian Arm Forces has been using this tool in the resilience and mental health training program to empower its members to self-care and monitor their own health, and observe signs of stress amongst their peers [55]. In the post-secondary context, the MHC tool can be used in educational sessions to promote dialogue about how mental health issues may be manifested on campus.

We have disseminated the tool and its finding of psychometric validation in a variety of ways within the universities that participated in the Caring Campus project [56]. For example, we provided it to student wellness services to be incorporated into mental health awareness activities. We disseminated the tool more widely within various mental health organizations’ workshops on campus as well, and used it in educational sessions to promote dialogue about the way mental health may be expressed within particular student groups. While the tool provides an a vehivle for health promotion activities in postsecondary environments, we need to understand better whether it can indeed be part of a strategy that helps students move from awareness to action, and improve their mental health.

Although postsecondary institutions are recognizing that they play a key role in maintaining the well-being of their students, the range and complexity of mental health issues among students cannot be addressed by the campuses alone. To facilitate more coordinated support for students, campuses need to develop alliances with social sectors, health sectors, and community-based agencies. Such off-campus relationships will facilitate better referrals of mental health resources and social support for students with complex mental health needs.

## Conclusions

In summary, Candian postsecondary college students are vulnerable to mental health problems. Easy access to a simple mental health assessment tool is indispensable for them to evaluate and monitor the fluidity of their mental health regularly. Our results showed that the MCH was a valid evaluation tool across three university settings in all six crucial dimensions to help students monitor signs and behavioral indicators of their mental health and take appropriate actions to improve it.

## Data Availability

The datasets used in this study are available from the corresponding author upon request.

## Abbreviations

MHC: Mental health continuum
K-10: The Kessler psychological distress scale
